# ^137^Cs-Based Variation of Soil Erosion in Vertical Zones of a Small Catchment in Southwestern China

**DOI:** 10.3390/ijerph16081371

**Published:** 2019-04-16

**Authors:** Jiacun Chen, Zhonglin Shi, Anbang Wen, Dongchun Yan, Taili Chen

**Affiliations:** 1 Institute of Mountain Hazards and Environment, Chinese Academy of Sciences, Chengdu 610041, China; chenjiacunzhangrui@163.com (J.C.); wabang@imde.ac.cn (A.W.); yandc@imde.ac.cn (D.Y.); chentaili1994@126.com (T.C.); 2 University of Chinese Academy of Sciences, Beijing 100049, China

**Keywords:** variation, soil erosion, fallout radionuclide ^137^Cs, Hengduan Mountains

## Abstract

The study of the variability of soil erosion in mountainous areas provides the basis for soil and water conservation work and forest ecological construction in a targeted way. In this study, Liangshan Town catchment, a typical catchment in the Hengduan Mountains region, southwest China, was selected to investigate the variation of soil erosion in different vertical zones using the ^137^Cs tracing technique. The mean ^137^Cs reference inventories varied between 573.51 and 705.54 Bq/m^2^, with the elevation increasing from 1600 to 2600 m. The rates of soil erosion exhibited a significant variation. Under the same land cover condition, the average annual soil erosion modulus of high-elevation forest (elevation > 2200 m) was 400.3 t/(km^2^·a). However, the average annual soil erosion modulus of a low-elevation sparse forest (elevation < 1600 m) was as high as 1756 t/(km^2^·a). The average annual soil erosion modulus of the sloping farmland, mainly distributed at elevations of 1600–2200 m, was estimated to be 2771 t/(km^2^·a). These results indicate that effective soil management measures need to be implemented on the cultivated sloping land in the future.

## 1. Introduction

Soil erosion remains one of the most important environmental problems that threaten the survival and sustainable development of human beings [1,2]. Concerns for the environmental problems associated with soil erosion in agricultural and forest land also directed attention to the impact of soil and water loss in mountainous and sloping farmland, where the soil is more exposed to intense erosion and the sediments from soil erosion have a great effect on the river system [3,4]. Therefore, understanding the key zones of soil and water imbalance is an important requirement [5].

Accelerated soil erosion and degradation caused by excessive land use make soil one of the scarce resources in both developed and developing countries. Therefore, quantitative assessments of soil erosion and the evaluation of the rate of soil loss are urgently needed for effective soil and water conservation measures and sediment management strategies. Despite an apparent progress in the quantification of soil losses and sediment redistribution achieved in China during the past several decades [6,7], an objective knowledge about the intensity of these processes is still insufficient in different landscapes, for example, the Hengduan Mountains area in southwest China [8]. The soils in this region are extremely prone to erosion due to the loose sedimentary rock structure and, especially, the intensive agricultural activities [9]. On-site effects of erosion include soil degradation and the associated crop productivity reduction in this region, while off-site problems include watercourse and reservoir siltation and the eutrophication of water bodies. Previous studies [10,11] have demonstrated that the Hengduan Mountains region is one of the main sources of sediment input to rivers such as the Lancang and Yangtze Rivers.

Fallout radionuclides (FRNs), such as ^137^Cs, ^210^Pb_ex_, and ^7^Be, have been widely used for assessing the rates of soil loss at different and temporal scales [12,13]. Anthropogenic fallout radionuclide ^137^Cs is the most commonly used radionuclide, and its deposition rate on the earth’s surface showed a significant peak in 1963 [14,15]. ^137^Cs is mainly absorbed by fine soil particles, especially clay minerals, after reaching the ground through dry and wet deposition [16]. It moves slowly in the vertical direction along water seepage and migrates mechanically through physical processes such as the erosion, transport, and deposition of soil particles in space [17,18]. ^137^Cs has proved to be a cost- and time-effective tool to evaluate soil redistribution due to erosion within the landscape and can complement the information provided by conventional erosion measurements and modelling [19,20]. This study aimed to quantify the variation of soil erosion and to explore the hotspots of soil loss on a small agricultural catchment of the Hengduan Mountains region, southwest China.

## 2. Materials and Methods

### 2.1. Study Area

**Figure 1:**
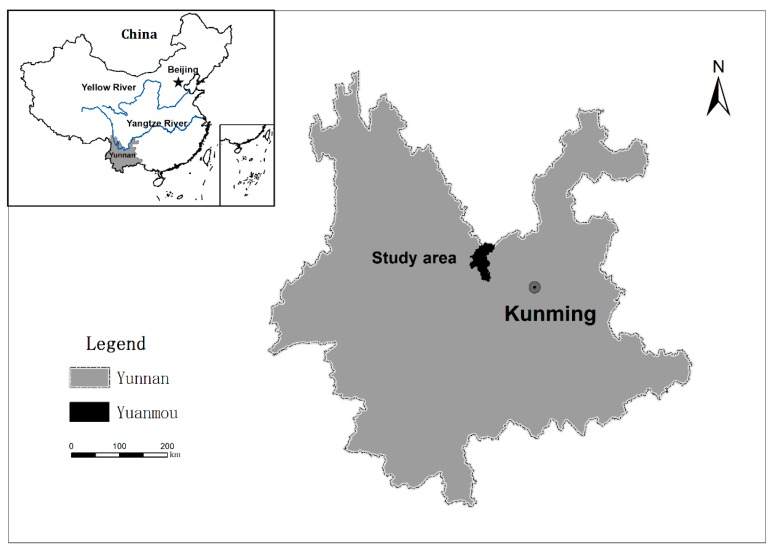
The geographical location of the study area.

**Figure 2:**
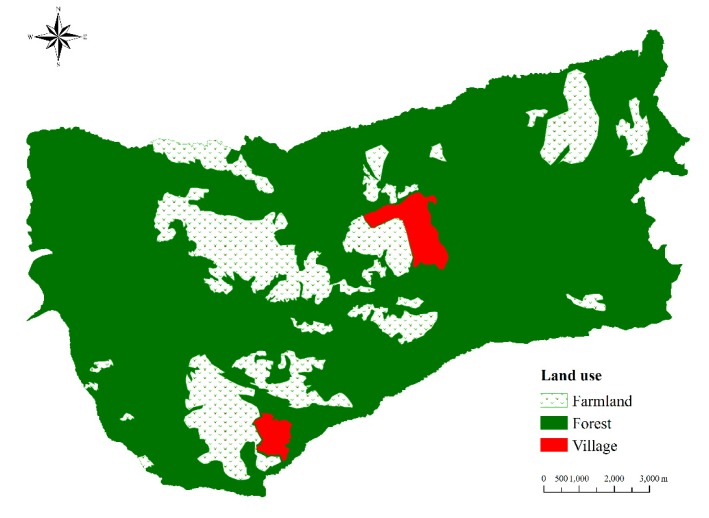
The land use types in Liangshan Town catchment.

The Liangshan Town catchment (4.4 km^2^), located in the Hengduan Mountains region in Yuanmou County of Yunnan Province (Figure 1), was chosen as the study area. The mean annual temperature and precipitation in the study area were 10.5 °C and 914.9 mm, respectively. The main soil types are yellow-brown soil and purple soil with bulk densities ranging from 1.2 to 1.4 g/cm^3^. The dominant land uses in this catchment area are forestry and cropland, accounting for 80.2% and 18.4% of the total area, respectively (Figure 2).

The forestland can be divided into two sections according to their elevations, namely the high-elevation forestland (2200–2835 m) and the low-elevation sparse forestland (1350–1600 m). In the former section, the forest is mainly composed of *Chir pine*, *Pinus yunnanensis,* and broadleaf tree species (*Quercus semecarpifolia* and *Quercus glauca*). However, in the low-elevation sparse forestland section, vegetation is dominated by *Dodonaea viscosa* shrubs and secondary eucalyptus [21].

The mid-catchment, with an elevation varying between 1600 and 2200 m, is characterized by cropland in terms of land use. Here, slope-cultivated land occupies 42.3% of the cropland area, followed by terrace land that occupies 35.6% and paddy fields that cover 22.1% of the cropland area.

### 2.2. Soil Sampling and ^137^Cs Measurements

The collection of soil samples from the catchment for ^137^Cs analysis involved two separate sampling strategies. The first was designed to establish the ^137^Cs reference inventories and their depth distribution in the soil profile. In this case, reference locations with a minimum slope and terrace land were selected for each vertical zone (i.e., 1350–1600 m, 1600–2200 m, and 2200–2835 m). The reference sites were sampled using a grid design to collect 5–10 samples, including one depth incremental sample for each vertical section. The sampling depths were 20–30 cm for forested sites and 30–40 cm for terrace land. The depth incremental samples were sectioned at 2-cm intervals. The second sampling strategy involved a coring program to establish the magnitude of soil redistribution rates within the catchment. In this case, the catchment was divided into several sampling units based on land use types (i.e., forest or cropland), and a minimum of 10 samples were taken for each unit. All samples were collected using a 7-cm diameter stainless steel coring device.

All samples were air-dried, disaggregated, and passed through a 2-mm sieve prior to a determination of their ^137^Cs activity by gamma spectrometry using a hyperpure lithium-drifted germanium detector (ORTEC, Oak Ridge, TN, USA). A sample weight of approximately 250 g and a counting time of over 35,000 s provided results with an analytical error of approximately ± 6% at the 95% level of confidence.

### 2.3. Conversion Models

A simplified Mass Balance Model [22] is widely used for the assessment of erosion rates on cultivated land, and it is expressed as follows:

(1) $$A=A_{0}{\left(1-h/H\right)}^{N-1963}$$

where *A*_0_ is the ^137^Cs reference inventory (Bq·m^−2^); *A* is the ^137^Cs inventory at an eroding point (Bq·m^−2^); *h* is the annual soil loss in depth since the year 1963 (cm); *H* is the plough depth (cm); and *N* is the sampling year.

For uncultivated soil, the depth distribution of ^137^Cs in the soil profile is significantly different from that in cultivated soils. Usually, the depth distribution of ^137^Cs in an undisturbed stable soil exhibits a well-defined exponential decline with depth, described by a Profile Distribution Model:

(2) $$A_{x}=A_{0}\left(1-e^{-x/h_{0}}\right)$$

where *x* is the mass depth from the soil surface (kg·m^−2^); *A_x_* is the ^137^Cs inventory above depth *x* (Bq·m^−2^); and *h_0_* is the coefficient describing the profile shape (kg·m^−2^).

## 3. Results

### 3.1. Variation of ^137^Cs Reference Inventory

The regional variability of ^137^Cs reference inventories in China were mainly related to rainfall and climate, China’s nuclear test in Xinjiang, and the former Soviet Union’s nuclear test in Central Asia [23]. Many studies have reported that ^137^Cs reference inventories in Yunnan province are rather low; the main reasons could be that this region is far away from the nuclear test sites as they are separated by mountains. In addition, because of the influence of the southwest monsoon climate, water vapours mainly come from the low latitude Indian Ocean, where few nuclear tests were conducted.

The moisture-rich monsoon airflow from the Indian Ocean form precipitation when forced to rise upon encountering high mountains. As vertical gradients of elevation in the study area reach more than 1500 m, the precipitation increases with elevation, and the distribution of precipitation varies significantly because of the large fluctuation of terrain. The elevation gradients of the catchment selected in this study range from 1350 m to 2835 m along the valley to the summit. The precipitation in the past two years increased from 652.2 to 914.9 mm, increasing by 40.3% along the valley to the top of the mountain. The ^137^Cs reference inventories increased by 23%, from 573.5 to 705.5 Bq/m^2^ along a 1600–2600-m elevation. The vertical variability of ^137^Cs reference inventories demonstrated that it was inaccurate to calculate the soil erosion modulus using only one mean value of reference inventories in the whole study area when the elevation and precipitation changed significantly in the vertical, when using the fallout radionuclide ^137^Cs technique to estimate soil erosion or sediments deposition. In contrast, the design of multilocation and multi-reference inventories was a more reasonable sampling method (Table 1).

American scientist Sutherland [24] pointed out that the sampling method and sample size are primary factors that affect the experimental results of the fallout radionuclide ^137^Cs technique. However, the sampling method and size of reference inventories are not described in detail in many studies that have applied this method. For reference areas with information on the number of control locations and the estimate of dispersion, the minimum number of samples (*n*’) necessary to estimate the mean ^137^Cs baseline inventory with an allowable error of 10% at a 90% confidence was determined using the following equation [25]:

(3) $$n^{\prime }={\left[\frac{t_{\left(a,n-1\right)}\cdot CV}{AE}\right]}^{2}$$

where *t* is the Student’s *t*-value for *a* = 0.10 (90% confidence), with *n* − 1 degrees of freedom. CV is the coefficient of variation (decimal fraction) and is defined as the standard deviation or arithmetic mean, and *AE* is the allowable error 0.1.

Thus, the experimental results are not convincible if it lacks details of the sample method and size of reference inventories, as the estimation of the soil erosion was based on the average value of the reference inventories. Unscientific reference inventories would increase the deviation between the estimated results and the actual values of soil erosion or sediment deposition. Therefore, the dispersion degree of data at the sampling location is a prerequisite to calculate the CV value of the data. The minimum sample size *n* at a 90% confidence interval under the *T* test is calculated based on the CV value, and the sampling of reference inventories is unrepresentative when the number of samples (*n*) is less than *n*’. The variation coefficients of ^137^Cs reference inventories at three vertical zones were all within the normal range in this study, with *n* > *n*’.

**Figure 3:**
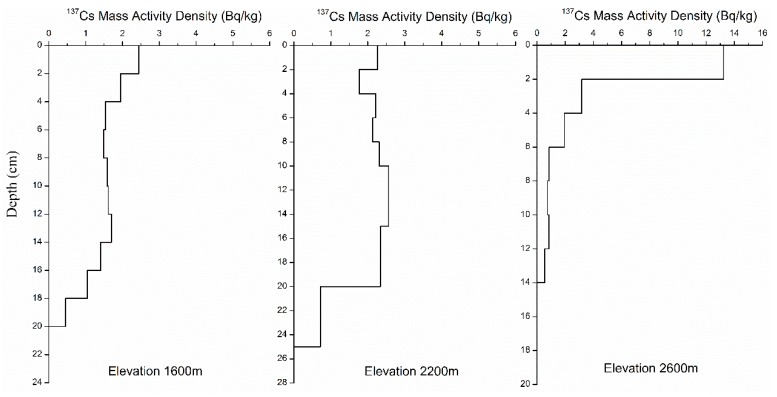
The depth distribution of ^137^Cs reference inventories (where 1600 m, 2200 m, and 2600 m represent the elevations of ^137^Cs reference locations).

Figure 3 showed the depth distribution of ^137^Cs reference inventories at the three sampling locations. The ^137^Cs reference inventories obtained under the *Pinus yunnanensis* forest at 2600 m, along a depth distribution, showed a significant variation in the exponential function because this site is covered with the Yunnan pine and is free from human interference due to its high elevation. The ^137^Cs activities of surface soil were the highest and reached up to 13.2 Bq/kg. The ^137^Cs activity values decreased exponentially with an increasing soil depth up to a 14-cm depth. The curve presented a typical depth variation of ^137^Cs in uncultivated soil, which indicated that the sampling sites had not been disturbed by human activities in the past 50 years. The depth distribution of ^137^Cs reference inventories obtained at 2200 m and 1600 m varied in a pattern typical of cultivated land. The ^137^Cs activity values in the agricultural layer (approx. 15 cm) were uniform and decreased dramatically below the agricultural layer. It was confirmed that the two reference locations were old terraced fields that had not been changed artificially in the past 50 years; these locations were suitable as reference sites for ^137^Cs at this elevation and were identified after inquiring with local farmers and investigations.

### 3.2. Variability of Soil Erosion Modulus in Different Vertical Zones

**Figure 4:**
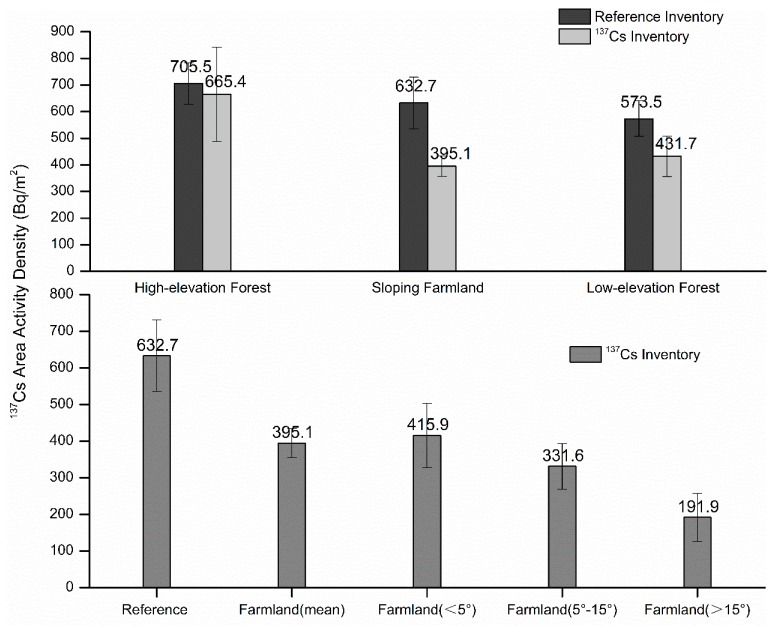
The variability of the reference inventory and the area activity density of ^137^Cs under different land use types.

**Figure 5:**
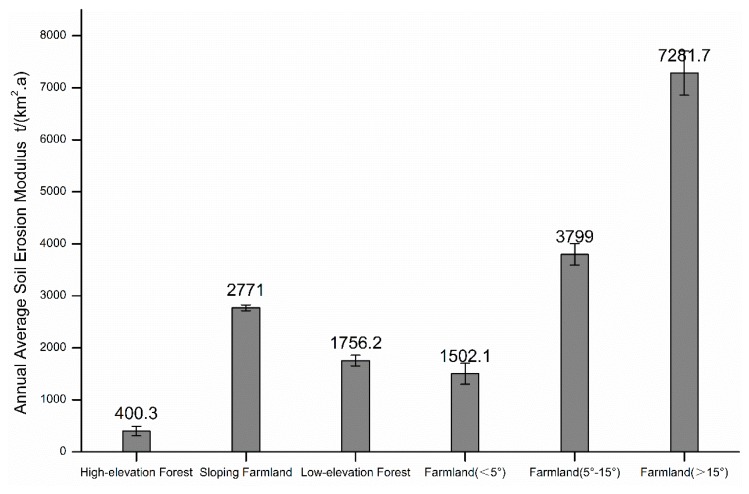
The soil erosion modulus under different land use types.

The average annual soil erosion modulus was calculated by comparing the ^137^Cs inventory values under different land use types and the ^137^Cs reference inventory in the corresponding vertical zones. The greater the difference between the ^137^Cs area activity density and the ^137^Cs reference inventory value, the more intense the soil erosion of the slope soil was (Figure 4). The average annual soil erosion modulus of a high-elevation forest (elevation > 2200 m) was 400.3 t/(km^2^·a), while that of a low-elevation sparse forest (elevation < 1600 m) was as high as 1756 t/(km^2^·a) with less annual rainfall. The average annual soil erosion modulus of a sloping farmland was 2771 t/(km^2^·a), which was mainly distributed between 1600 and 2200 m. Thus, the value of 632.7 Bq/m^2^ was used as the reference inventory in the calculation process. The steep slope farmland (slope > 15°) had an intense soil erosion, with an average annual soil erosion modulus as high as 7282 t/(km^2^.a) (Figure 5).

Many researchers have used different methods to study soil erosion in the Hengduan Mountains. For example, Wen [26] obtained an average soil erosion modulus of 1668 t/(km^2^·a) in the upper reaches of Longchuan River, based on the relationship between the sediment transport modulus and catchment area. In this study area, the Liangshan Town catchment, the average soil erosion intensity of a sloping farmland, was 2771 t/(km^2^·a) over the recent decades, which belonged to moderate erosion intensity; the average soil erosion intensity of a sparse forestland was 1756 t/(km^2^·a), which belonged to a mild erosion intensity, indicating that the soil erosion situation in this area was not serious.

The soil erosion modulus of an entire catchment can be calculated using the following formula [27]:

(4) Ew=(∑i=1nSi·Ei)Stot

where *E_w_* is the erosion modulus for the entire catchment or area (t·km^−2^·a^−1^); *n* is the number of sampling units; *S_i_* is the surface area of the sampling units; *S_tot_* is the surface area of the entire catchment or area (km^2^); and *E_i_* is the average erosion modulus of the representative fields of the sampling unit *i* (t·km^−2^·a^−1^). The average soil erosion modulus of the entire catchment was 1216.5 t/(km^2^·a), calculated using Equation (4), which was lower than the values obtained by other researchers in this region (Table 2).

## 4. Discussion

### 4.1. Factors Influencing the Local ^137^Cs Reference Inventory

The region’s representative reference inventory of fallout radionuclide ^137^Cs was the basis for using the ^137^Cs technique to estimate the average soil erosion or the deposition rate in recent decades [28]. The reference inventories of radionuclides were significantly different from each other at different longitudes and elevations in the world [29]. Because of the combined actions of different factors, the reference inventories of radionuclides in the same basin still showed a variability, mainly affected by the average annual precipitation, slope, and land use pattern within small catchments [30].

The variability of ^137^Cs reference inventories were presented as (1) the differences in ^137^Cs reference inventories between different latitudes and longitudes and between the southern and northern hemispheres, which showed a close relationship with the geographical locations where nuclear tests were conducted in the last century [31]; (2) a variability at different elevations of the same latitude and longitude, with the change in elevation also being the main factor inducing variation in annual precipitation. In areas where wet precipitation takes the leading role, the average annual precipitation was the key factor influencing the ^137^Cs reference inventories. Related research [32,33] demonstrated that the correlation coefficient of the variation in annual precipitation and the ^137^Cs reference inventories is 0.5; (3) there were significant differences in the reference inventories under different land use types [34]. Related studies indicated that grassland was the most suitable land cover type that reflected the real background value of the region. Normally, flat grassland was the optimum land cover type for sampling ^137^Cs reference inventories due to its small slope and runoff and slight disturbance level. In forestland, affected by canopy interception, ^137^Cs reference inventories near tree trunks were higher than that under the canopy, which results in a variation of ^137^Cs reference inventories within the forestland [35]. In addition, the main roots of woodland trees were mainly distributed in the surface soil and caused ab inconvenience for sample collection. Under these circumstances, the terraced fields that had been abandoned for decades or fields that have not been eroded were often considered as sampling sites for reference inventories.

### 4.2. Causes of Soil Erosion Within Vertical Zones and Soil Conservation Measures

The variability of soil erosion showed an obvious vertical zonality. The soil erosion intensity in low-elevation zones was obviously higher than that in high-elevation zones under the same land cover type. ^137^Cs is mainly absorbed by fine soil particles, especially clay mineral, after reaching the ground. Although the soils in high-elevation zones are dominated by Yellow-brown soils and those in low-elevation zones are dominated by purple soils, there is no significant difference in the clay content between the two soil types [21]. The main reason was that annual rainfall in high-elevation zones was 40% more than that in low-elevation zones, which led to sufficient soil moisture and high vegetation coverage in high-elevation zones. In addition, the temperature decrease with an increasing elevation and low annual evapotranspiration, which enabled the surface soil to maintain a certain amount of water under very low precipitations in winter, supported the normal growth of plants at high-elevation zones. A high vegetation cover and a complete forest ecosystem were the key factors reducing soil erosion. Because of the special monsoon climate and less rainfall, a high temperature and drought occurred in winter in the low-elevation zones, accompanied by large soil evapotranspiration. As a result, the surface vegetation coverage rate was low and the vegetation was mainly low drought-tolerant shrubs, with fewer herbs and a large area of bare soil. When the summer rainstorm was concentrated, surface erosion and rill erosion were serious.

A sloping farmland had the greatest soil erosion in the catchment. Soil erosion on sloping farmlands was the main source of sediments in the river. The average soil erosion intensity of a sloping farmland in the Liangshan Town catchment was 2771 t/(km^2^·a), which was less than the values obtained by other researchers in this region. This was mainly due to the gentle slope of the farmland, which was terraced mainly in the Liangshan Town catchment; the farmland that had been effectively maintained by local farmers; the export of rural labour forces in recent decades; and the effect of returning farmland to forest (pasture).

The results of the present study in Liangshan Town catchment showed that the sloping farmland in the mountainside zone and the sparse woodland in the low-elevation zone were the key zones of the soil and water imbalance in Hengduan Mountains, southwest China. Reducing steep the slope tillage, introducing more cash crops and fruit trees, and adjusting the agricultural structure were the effective approaches to reducing soil erosion in the region. In order to control soil erosion in the low-elevation sparse forest zone, the best measures were introducing drought-resistant forest trees and grasses and gully control, such as by planting drought-resistant pioneer plants mainly composed of *Gladiolus* and *Leucaena leucocephala*, which can improve the soil-vegetation system structure and change the microclimate. Finally, other economically beneficial trees could be introduced.

In general, steep-sloping farmland and low-elevation sparse forestland should be taken as key areas of soil and water imbalance and effectively harnessed according to local conditions for soil and water conservation in the Hengduan Mountains of southwest China. Although the two soil types of yellow-brown and purple soils have comparable clay contents as indicated above, other properties (e.g., organic matter content and pH) that have the potential to affect the adsorption of ^137^Cs in soils have not been measured in present work, which should be seen as a limitation of this study. Further works are needed to focus on these issues to strengthen the successful use of the ^137^Cs method in this area.

## 5. Conclusions

In this study, anthropogenic fallout radionuclide ^137^Cs was used to investigate the variability of soil erosion in Liangshan Town, a small agricultural catchment in Hengduan Mountains. Three ^137^Cs reference locations along the vertical zones from the summit to the valley were established according to differences in the elevation and rainfall. The results showed that the reference inventory of ^137^Cs increased from 573.51 Bq/m^2^ to 705.54 Bq/m^2^ with the elevation rising from 1600 m to 2600 m, indicating a significant variability. In order to accurately calculate the soil erosion modulus of different land-use types under different vertical zones, the ^137^Cs inventory values in each vertical zone were compared with the ^137^Cs reference inventory in the corresponding vertical zone. The results showed that a sloping farmland had the most intense soil erosion, making sloping farmland one of the important sources of sediments in the catchment. The average soil erosion intensity of a sloping farmland in this catchment was 2771 t/(km^2^·a), which belonged to the category of moderate erosion according to the classification of soil erosion intensity. The average annual soil erosion modulus was as high as 7282 t/(km^2^·a) when the slope of the cultivated land was greater than 15 degrees, which belong to an intense erosion category. The average annual soil erosion modulus of a low-elevation sparse forest zone (elevation < 1600 m) was 1756 t/(km^2^·a), which is significantly higher than that of the high-elevation forest zone (elevation > 2200 m) with a value of 400 t/(km^2^·a).

## Figures and Tables

**Table 1:** The background of the ^137^Cs reference inventory sampling location at different vertical zones.

Elevation (m)	Rainfall (mm)	Soil Type	Land Use	Actual Sample Size (*n*)	CV (%)	Minimum Number of Samples (*n*’)	^137^Cs Reference Inventory (Bq/m^2^)
2600	914.9	Yellow-brown	Forest	5	11.06	4.96	705.54
2200	783.8	Purple soil	Terrace	10	15.41	7.97	632.68
1600	652.2	Purple soil	Terrace	6	11.65	5.51	573.51

**Table 2:** The soil erosion modulus of the Liangshan Town catchment.

Land Use Types	High-Elevation Forestland	Sloping Farmland	Low-Elevation Forestland	Total
Surface area (km^2^)	2.28	0.81	1.25	4.4
Average erosion modulus (t·km^−2^·a^−1^)	400.3	2771	1756.2	1216.5

The surface area of the village was 0.06 km^2^, and the soil erosion of the village was neglected.
